# Importance of Aggregated Islet Amyloid Polypeptide for the Progressive Beta-Cell Failure in Type 2 Diabetes and in Transplanted Human Islets

**DOI:** 10.1155/2008/528354

**Published:** 2009-03-31

**Authors:** Gunilla T. Westermark, Per Westermark

**Affiliations:** ^1^Division of Cell Biology, Diabetes Research Centre, Department of Clinical and Experimental Medicine, Linköping University, 58185 Linköping, Sweden; ^2^Department of Medical Cell Biology, Uppsala University, 75123 Uppsala, Sweden; ^3^Department of Genetics and Pathology, Uppsala University, 75185 Uppsala, Sweden

The almost constantly appearing amyloid deposits in islets of Langerhans of individuals with type 2 diabetes was for long time regarded as more or less innocent bystanders. Even after that the amyloid was shown to be an aggregated form of a novel polypeptide hormone, islet amyloid polypeptide (IAPP or amylin), the deposits themselves attracted little interest. This can appear peculiar today and is in sharp contrast to another localized form of amyloid deposition occurring in the brain particularly in association with Alzheimer's disease. The protein forming the brain amyloid, A*β*-peptide, was discovered just two years (1984) before IAPP (1986), and aggregation of A*β* became immediately a central issue in the studies of the pathogenesis of Alzheimer's disease. So, why did almost the whole research field on senile and presenile dementia so rapidly focus on amyloid while most diabetologists, with a few exceptions, disregarded islet amyloid? A possible reason is that while Alzheimer researchers fumbled after a possible pathogenic mechanism for the brain pathology, the research field of type 2 diabetes was already established and in a high degree directed towards the development of insulin resistance. Many researchers regarded the obvious failure of beta-cells only as a secondary event due to some elusive mechanism of “glucose toxicity.” In addition, experimentalists in diabetes research most often used mice or rats as models, and in these species, islet amyloid cannot develop due to the amino acid sequence of their IAPP molecules.

After the discovery of IAPP, there was a strong interest in the possible physiological role of the molecule and in the effect it may have on the development of type 2 diabetes. It was found early that the peptide induces insulin insensitivity in peripheral tissues. However, this effect was reached only after nonphysiological levels of IAPP. More established effects of the peptide include regulation of satiety, gastric emptying and para- or autocrine signalling of insulin, and glucagon secretion. All these effects seem to modulate the action of insulin, leading to a more even blood glucose level. Since IAPP is a beta-cell product and these cells are lost in type 1 diabetes, IAPP in a modified form has been introduced as supplement to insulin treatment.

Today, however, we can note an increasing interest in the importance of the development of IAPP-derived islet amyloid on the beta-cell function. Earlier it was believed that islet amyloid could not be of any significance since even in cases with pronounced deposits, there are always well-granulated beta-cells left. There are two essentially new findings that are of particularly great interest. First, islet amyloid may initially assemble intracellularly with severe consequences for the affected cell. Second, there is growing evidence that small, oligomeric, prefibrillar aggregates of IAPP are directly toxic to beta-cells. Interestingly, there is also increasing evidence that this reflects a generic mechanism common to many protein aggregates in which beta-sheet formation are essential. Such aggregates of often completely nonrelated peptides seem to have similar or identical effects on cells. It is still very incompletely understood which these effects are. Eventually, the interactions may lead to cell death, and aggregated IAPP is a strong candidate as an important cause of beta-cell loss in type 2 diabetes. Cell death and accumulation of diffuse amyloid deposits within the islet of Langerhans are sufficient to destroy the islet architecture which is important for maintaining optimal signaling.

The finding that isolated normal human islets, transplanted into nude mice, very rapidly develop IAPP-derived amyloid deposits intra- and extracellularly, lead to the suspicion that such an event could be an important cause of the loss of function of islets transplanted into type 1 diabetic individuals. This hypothesis was recently strongly supported by the analysis of a liver of a deceased person, who had received intraportal islet transplants on three occasions. Almost 50% of identified islets were found to contain amyloid deposits. Obviously, this may mean that a beta-cell lesion, typical of type 2 diabetes, may develop in originally normal islets transplanted into a type 1 diabetic individual. Methods to inhibit amyloid formation may be important to develop in order to prolong the survival of transplanted islets.

The present issue of Experimental Diabetes Research deals with different aspects of islet amyloid and IAPP. We hope that the papers will lead to further interest in this field which sometimes has been neglected in diabetes research.

*Gunilla T. Westermark* *Gunilla T. Westermark* *Per Westermark* *Per Westermark*

